# Supplementary material for: Network spreading and local biological vulnerability in amyotrophic lateral sclerosis
Source: Commun Biol. 2025 Aug 4;8:1153. doi: 10.1038/s42003-025-08561-3 (PMC12322078; doi:10.1038/s42003-025-08561-3)
Supplement: Supplementary file 1 — Supplementary Information [file 42003_2025_8561_MOESM1_ESM.pdf]

Supplementary Information

The S.I.R model

The S.I.R model has been previously used to explore the spreading of pathological proteins in Parkinson disease [1, 2], prodromal synucleinopathy [3], and frontotemporal dementia [4]. This section briefly summarizes the model's main equations.

The initial step of this model consists in determining the baseline regional density of some protein of interest in individual parcels of the network. To do so, we increment the population of normal agents (i.e., proteins) in region i, N_i, with

$$\Delta N_i = \alpha_i S_i \Delta t - (1 - e^{-\beta_i \Delta t}) N_i \tag{1}$$

where α_i and β_i are respectively the synthesis and clearance rate of the protein in region i. S_i corresponds to the size (vertex count) of region i and Δt is the time interval between two iterations, which is set to 0.02.

After the system reaches the stable point (error tolerance $\epsilon < 10^{-7}$), the pathogenic spread of misfolded proteins is initiated and the population of normal (N) and misfolded (M) agent is updated with

$$\begin{aligned}\Delta N_i =& \alpha_i S_i \Delta t - (1 - e^{-\beta_i \Delta t}) N_i \\ &- (e^{-\beta_i \Delta t})(1 - e^{-\gamma_i^0 M_i \Delta t}) N_i\end{aligned} \tag{2}$$

$$\begin{aligned}\Delta M_i =& (e^{-\beta_i \Delta t})(1 - e^{-\gamma_i^0 M_i \Delta t}) N_i \\ &- (1 - e^{-\beta_i \Delta t}) M_i\end{aligned} \tag{3}$$

where γ_i^0 is the baseline transmission rate that measures the likelihood that a single misfolded agent can transmit the infection to other susceptible agents. This baseline transmission rate was set to $1/S_i$.

For each iteration, agents in region i may remain in the same region or may enter one of its edges according to a multinomial distribution with probabilities

$$P_{\text{region}i \to \text{region}i} = \rho_i \tag{4}$$

$$P_{\text{region}i \to \text{edge}(i,j)} = (1 - \rho_i) \frac{w_{ij}}{\sum_j w_{ij}} \tag{5}$$

where w_{ij} is the connection strength of edge (i, j). The probability of remaining in the current region, ρ_i, was set to 0.99. Analogously, the agents in edge (i, j) may exit the edge or remain in the same edge per unit of time with binary probabilities:

$$P_{\text{edge}(i,j) \to \text{region}(j)} = \frac{1}{l_{ij}} \tag{6}$$

$$P_{\text{edge}(i,j) \to \text{edge}(i,j)} = 1 - \frac{1}{l_{ij}} \tag{7}$$

where l_{ij} is the length of edge (i, j).

We use $N_{i,j}$ and $M_{i,j}$ to denote the normal/misfolded population in edge (i, j). For each interval of time Δt, the increments of N_i, M_i in region i are

$$\Delta N_i = \sum_j \frac{1}{l_{ji}} N_{(j,i)} \Delta t - (1 - \rho_i) N_i \Delta t \tag{8}$$

$$\Delta M_i = \sum_j \frac{1}{l_{ji}} M_{(j,i)} \Delta t - (1 - \rho_i) M_i \Delta t \tag{9}$$

Likewise,

$$\Delta N_{(i,j)} = (1 - \rho_i) \frac{w_{ij}}{\sum_j w_{ij}} N_i \Delta t - \frac{1}{l_{ij}} N_{(i,j)} \Delta t \tag{10}$$

$$\Delta M_{(i,j)} = (1 - \rho_i) \frac{w_{ij}}{\sum_j w_{ij}} M_i \Delta t - \frac{1}{l_{ij}} M_{(i,j)} \Delta t \tag{11}$$

Finally, we model neuronal tissue loss (L) as the result of two processes: direct toxicity from accumulation of native misfolded proteins and deafferentation (reduction in neuronal inputs) from neuronal death in neighbouring (connected) regions. The atrophy accrual at t within Δt in region i is given by the sum of the two processes:

$$\begin{aligned}\Delta L_i =& k_1 (1 - e^{r_i(t) \Delta t}) \\ &+ k_2 \sum_j \frac{w_{ij}}{\sum_j w_{ij}} (1 - e^{-r_j(t-1) \Delta t})\end{aligned} \tag{12}$$

Where $r_i(t)$ is the proportion of misfolded agents in region i at time t. k_1 and k_2 are both set to 0.5 such that the two processes have equal importance in modeling the total atrophy growth. A list of the free parameters that have been used can be found in Table. S3.

Gene enrichment details

All explanations in this section are sourced from the European Bioinformatics Institute website (https://www.ebi.ac.uk). Below, we provide detailed descriptions for the terms presented in Fig. 4a, under the section "GO-BiologicalProcessDirect".

- **GO:0001508 - Action potential**: A process in which membrane potential cycles through a depolarizing spike, triggered in response to depolarization above some threshold, followed by repolarization. This cycle is driven by the flow of ions through various voltage-gated channels with different thresholds and ion specificities.
- **GO:0006096 - glycolytic process**: The chemical reactions and pathways resulting in the breakdown of a carbohydrate into pyruvate, with the concomitant production of a small amount of ATP and the reduction of NAD(P) to NAD(P)H. Glycolysis begins with the metabolism of a carbohydrate to generate products that can enter the pathway and ends with the production of pyruvate. Pyruvate may be converted to acetyl-coenzyme A, ethanol, lactate, or other small molecules.
- **GO:0009083 - branched-chain amino acid catabolic process:** The chemical reactions and pathways resulting in the breakdown of amino acids containing a branched carbon skeleton, comprising isoleucine, leucine and valine.
- **GO:0006099 - tricarboxylic acid cycle**: A nearly universal metabolic pathway in which the acetyl group of acetyl coenzyme A is effectively oxidized to two CO^2 and four pairs of electrons are transferred to coenzymes. The acetyl group combines with oxaloacetate to form citrate, which undergoes successive transformations to isocitrate, 2-oxoglutarate, succinyl-CoA, succinate, fumarate, malate, and oxaloacetate again, thus completing the cycle. In eukaryotes the tricarboxylic acid is confined to the mitochondria.
- **GO:0032781 - positive regulation of ATP-dependent activity**: Any process that activates or increases the rate of an ATP-dependent activity.
- **GO:0090141 - positive regulation of mitochondrial fission:** Any process that increases the rate, frequency or extent of mitochondrial fission. Mitochondrial fission is the division of a mitochondrion within a cell to form two or more separate mitochondrial compartments.
- **GO:0016226 - iron-sulfur cluster assembly**: The incorporation of iron and exogenous sulfur into a metallo-sulfur cluster.
- **GO:0090383 - phagosome acidification**: Any process that reduces the pH of the phagosome, measured by the concentration of the hydrogen ion.
- **GO:0030150 - protein import into mitochondrial matrix**: The import of proteins across the outer and inner mitochondrial membranes into the matrix. Unfolded proteins enter the mitochondrial matrix with a chaperone protein; the information required to target the precursor protein from the cytosol to the mitochondrial matrix is contained within its N-terminal matrix-targeting sequence. Translocation of precursors to the matrix occurs at the rare sites where the outer and inner membranes are close together.
- **GO:0006094 - gluconeogenesis**: The formation of glucose from noncarbohydrate precursors, such as pyruvate, amino acids and glycerol.
- **GO:0050905 - neuromuscular process**: Any process pertaining to the functions of the nervous and muscular systems of an organism.
- **GO:0006851 - mitochondrial calcium ion transmembrane transport**: The process in which a calcium ion (Ca^{2+}) is transported across a mitochondrial membrane, into or out of the mitochondrion.
- **GO:0006446 - regulation of translational initiation:** Any process that modulates the frequency, rate or extent of translational initiation.
- **GO:0033572 - transferrin transport**: The directed movement of transferrin into, out of or within a cell, or between cells, by means of some agent such as a transporter or pore.
- **GO:0032418 - lysosome localization**: Any process in which a lysosome is transported to, and/or maintained in, a specific location.
- **GO:1990869 - cellular response to chemokine**: Any process that results in a change in state or activity of a cell (in terms of movement, secretion, enzyme production, gene expression, etc.) as a result of a chemokine stimulus.
- **GO:1990573 - potassium ion import across plasma membrane**: The directed movement of potassium ions from outside of a cell, across the plasma membrane and into the cytosol.
- **GO:1902600 - hydrogen transmembrane transport**: The directed movement of a proton across a membrane.
- **GO:0047496 - vesicle transport along microtubule**: The directed movement of a vesicle along a microtubule, mediated by motor proteins. This process begins with the attachment of a vesicle to

a microtubule, and ends when the vesicle reaches its final destination.

- **GO:0000422 - mitophagy (autophagy of mitochondrion)**: The autophagic process in which mitochondria are delivered to a type of vacuole and degraded in response to changing cellular conditions.
- **GO:0032402 - melanosome transport**: The directed movement of melanosomes into, out of or within a cell, or between cells, by means of some agent such as a transporter or pore.
- **GO:0030048 - actin filament-based movement**: Movement of organelles or other particles along actin filaments, or sliding of actin filaments past each other, mediated by motor proteins.
- **GO:0007588 - excretion**: The elimination by an organism of the waste products that arise as a result of metabolic activity. These products include water, carbon dioxide (CO^2), and nitrogenous compounds.
- **GO:0046034 - ATP metabolic process**: The chemical reactions and pathways involving ATP, adenosine triphosphate, a universally important coenzyme and enzyme regulator.
- **GO:0045780 - positive regulation of bone resorption**: Any process that activates or increases the frequency, rate or extent of bone resorption.
- **GO:0018146 - keratan sulfate biosynthetic process**: The chemical reactions and pathways resulting in the formation of keratan sulfate, a glycosaminoglycan with repeat units consisting of beta-1,4-linked D-galactopyranosyl-beta-(1,4)-N-acetyl-D-glucosamine 6-sulfate and with variable amounts of fucose, sialic acid and mannose units; keratan sulfate chains are covalently linked by a glycosidic attachment through the trisaccharide galactosyl-galactosyl-xylose to peptidyl-threonine or serine residues.
- **GO:0007628 - adult walking behavior**: The behavior of an adult relating to the progression of that organism along the ground by the process of lifting and setting down each leg.
- **GO:0006865 - amino acid transport**: The directed movement of amino acids, organic acids containing one or more amino substituents, into, out of or within a cell, or between cells, by means of some agent such as a transporter or pore.
- **GO:0006687 - glycosphingolipid metabolic process**: The chemical reactions and pathways involving glycosphingolipids, any compound with residues of sphingoid and at least one monosaccharide.
- **GO:0098662 - inorganic cation transmembrane transport**: A process in which an inorganic cation is transported from one side of a membrane to the other by means of some agent such as a transporter or pore.

Detailed descriptions for the terms found in Fig. 4b, under the section “GO-CellularComponentDirect” are listed below.

- **GO:0031305 - integral component of mitochondrial inner membrane**: The component of the mitochondrial inner membrane consisting of the gene products having at least some part of their peptide sequence embedded in the hydrophobic region of the membrane.
- **GO:0031307 - integral component of mitochondrial outer membrane**: The component of the mitochondrial outer membrane consisting of the gene products having at least some part of their peptide sequence embedded in the hydrophobic region of the membrane.
- **GO:0030673 - axolemma**: The portion of the plasma membrane surrounding an axon; it is a specialized trilaminar random mosaic of protein molecules floating within a fluid matrix of highly mobile phospholipid molecules, 7-8 nm in thickness.
- **GO:0042645 - mitochondrial nucleoid**: The region of a mitochondrion to which the DNA is confined.
- **GO:0005875 - microtubule associated complex**: Any multimeric complex connected to a microtubule.
- **GO:0031966 - mitochondrial membrane**: Either of the lipid bilayers that surround the mitochondrion and form the mitochondrial envelope.
- **GO:0097440 - apical dendrite**: A dendrite that emerges near the apical pole of a neuron. In bipolar neurons, apical dendrites are located on the opposite side of the soma from the axon.
- **GO:0005782 - peroxisomal matrix**: The volume contained within the membranes of a peroxisome; in many cells the matrix contains a crystalloid core largely composed of urate oxidase.
- **GO:0005759 - mitochondrial matrix**: The gel-like material, with considerable fine structure, that lies in the matrix space, or lumen, of a mitochondrion. It contains the enzymes of the tricarboxylic acid cycle and, in some organisms, the enzymes concerned with fatty acid oxidation.

- **GO:0005777 - peroxisome**: A small organelle enclosed by a single membrane, and found in most eukaryotic cells. Contains peroxidases and other enzymes involved in a variety of metabolic processes including free radical detoxification, lipid catabolism and biosynthesis, and hydrogen peroxide metabolism.
- **GO:0019897 - extrinsic component of plasma membrane**: The component of a plasma membrane consisting of gene products and protein complexes that are loosely bound to one of its surfaces, but not integrated into the hydrophobic region.
- **GO:0016235 - aggresome**: An inclusion body formed by dynein-dependent retrograde transport of an aggregated protein on microtubules.
- **GO:0031225 - anchored component of membrane**: The component of a membrane consisting of the gene products that are tethered to the membrane only by a covalently attached anchor, such as a lipid group that is embedded in the membrane. Gene products with peptide sequences that are embedded in the membrane are excluded from this grouping.
- **GO:0030659 - cytoplasmic vesicle membrane**: The lipid bilayer surrounding a cytoplasmic vesicle.
- **GO:0032587 - ruffle membrane**: The portion of the plasma membrane surrounding a ruffle.
- **GO:0055037 - recycling endosome**: An organelle consisting of a network of tubules that functions in targeting molecules, such as receptors transporters and lipids, to the plasma membrane.
- **GO:0019898 - extrinsic component of membrane**: The component of a membrane consisting of gene products and protein complexes that are loosely bound to one of its surfaces, but not integrated into the hydrophobic region.
- **GO:0031234 - extrinsic component of cytoplasmic side of plasma membrane**: The component of a plasma membrane consisting of gene products and protein complexes that are loosely bound to its cytoplasmic surface, but not integrated into the hydrophobic region.

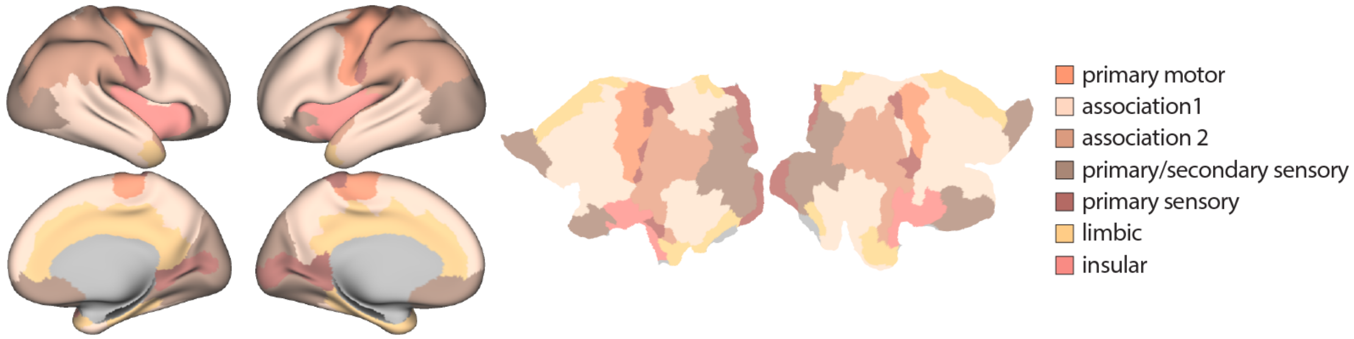

Figure S1. **Von Economo's cytoarchitectonic classes |** Von Economo cytoarchitectonic parcellation [5–7] is shown on the fs-LR inflated (left) and flat (right) cortical surfaces.

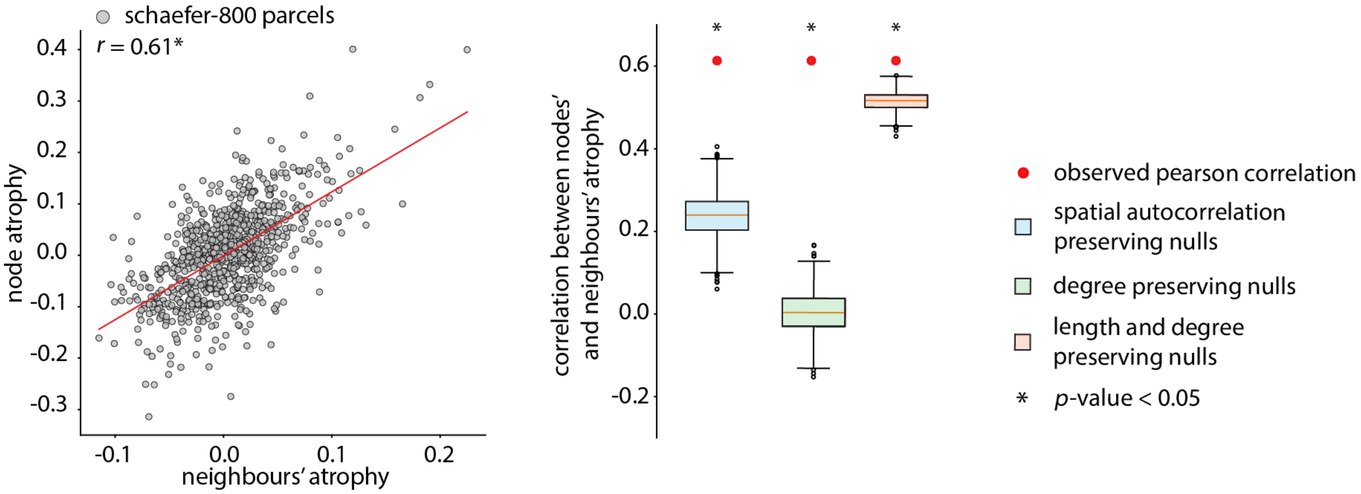

Figure S2. **Replication of ALS-related cortical atrophy dependence on structural connectivity using the Schaefer-800 parcellation |** Left: The scatter plot shows the atrophy of a node (y-axis) and the mean atrophy of that node's structurally connected neighbours (x-axis) (Pearson correlation coefficient; $r = 0.61$). Grey circles indicate brain regions defined in Schaefer-800 parcellation [8]. Right: The observed correlation coefficients between node and neighbour atrophy (red circles) are shown with respect to three null models: (1) spatial autocorrelation preserving spin tests ($p = 9.99 \times 10^{-4}$, $n_{\text{spin}} = 1,000$, blue box plot), (2) degree-preserving rewired networks ($p = 9.99 \times 10^{-4}$, $n_{\text{spin}} = 1,000$, green box plot), and (3) degree- and edge length-preserving rewired networks ($p = 9.99 \times 10^{-4}$, $n_{\text{spin}} = 1,000$, red box plot). Asterisks indicate statistical significance with respect to each null model.

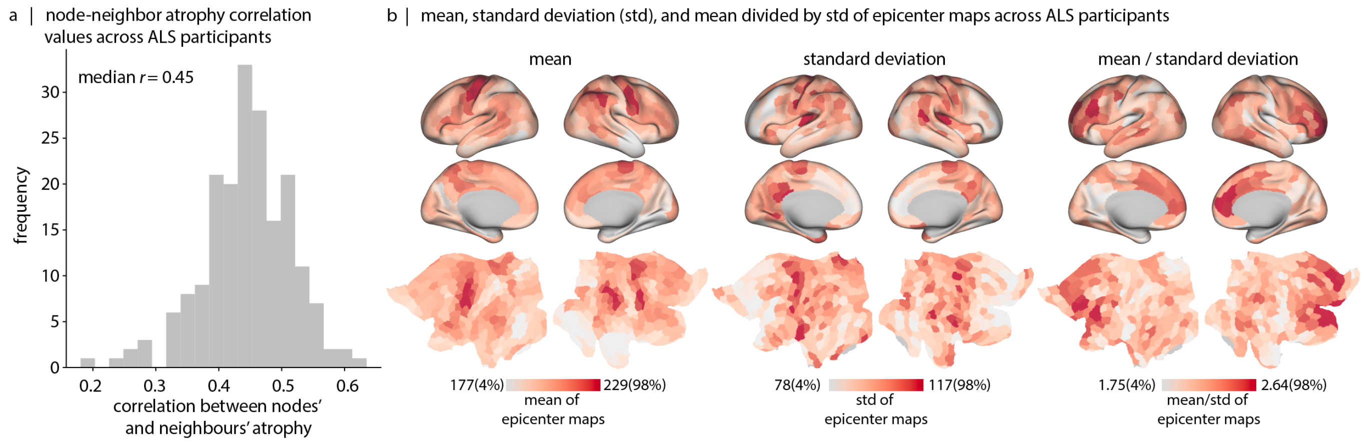

Figure S3. **Inter-individual differences in node-neighbour correlation values and epicenter maps across ALS participants |** Here the w-score map (after multiplying by -1) is used as a participant-specific atrophy map. (a) A histogram of node-neighbour correlation values, representing the correlation between a node and the mean atrophy of that node's structurally connected neighbours, is shown across ALS participants. These correlation values are derived using the consensus structural connectome of healthy young adults in the HCP dataset. The median of the individualized node-neighbour correlation values is $r = 0.45$. (b) For each ALS participant, an epicenter likelihood value is assigned to each cortical node using the atrophy ranking method (see *Methods*). After estimating the epicenter likelihood map per participant, the map is normalized so that the highest-probability epicenter parcel scores 1, and the lowest scores 0. We average these normalized maps across all ALS participants to produce a mean epicenter map. The standard deviation, and mean divided by standard deviation for each parcel is also calculated. The mean, standard deviation, and mean divided by standard deviation maps are shown on the fs-LR inflated and flat cortical surfaces.

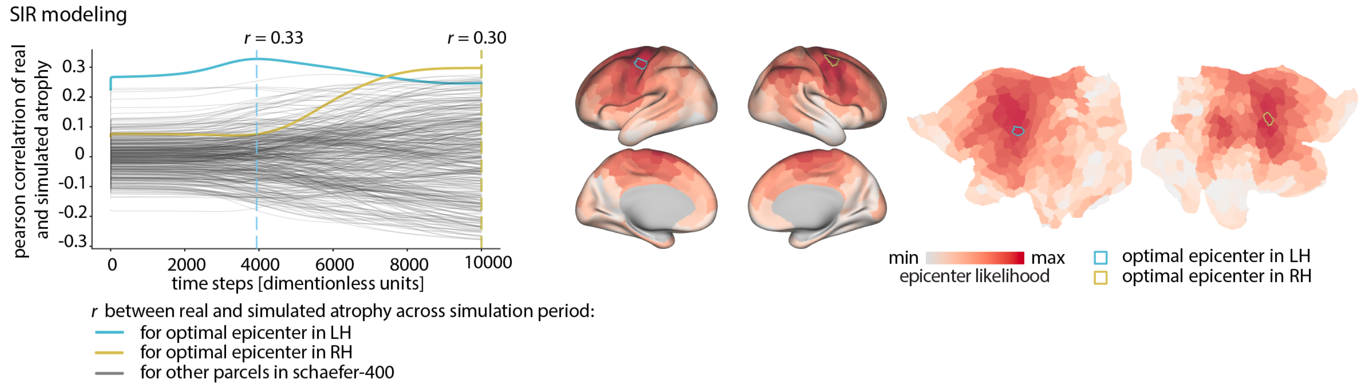

Figure S4. **ALS cortical epicenter likelihood map derived from the SIR modeling approach |** The Agent-based SIR model only considers the structural connectome as the underlying network for pathology spread. The spreading process is initiated in every brain region and the correlation between the simulated and empirical patterns of atrophy is computed at each simulation time point. The two largest correlations are obtained by seeding regions within the motor cortex (indicated by a blue border on the left cortical surface (LH) and by a yellow border on the right cortical surface (RH). The epicenter likelihood maps obtained by the SIR modeling approach are shown on both inflated and flat fs-LR cortical surfaces. The epicenter likelihood maps obtained via both the ranking method (Fig. 2c) and the SIR modeling lead to cortical patterns which are correlated with each other (Pearson correlation coefficient; $r = 0.77$, $p_{\text{spin}} = 9.99 \times 10^{-4}$, $n_{\text{spin}} = 1,000$).

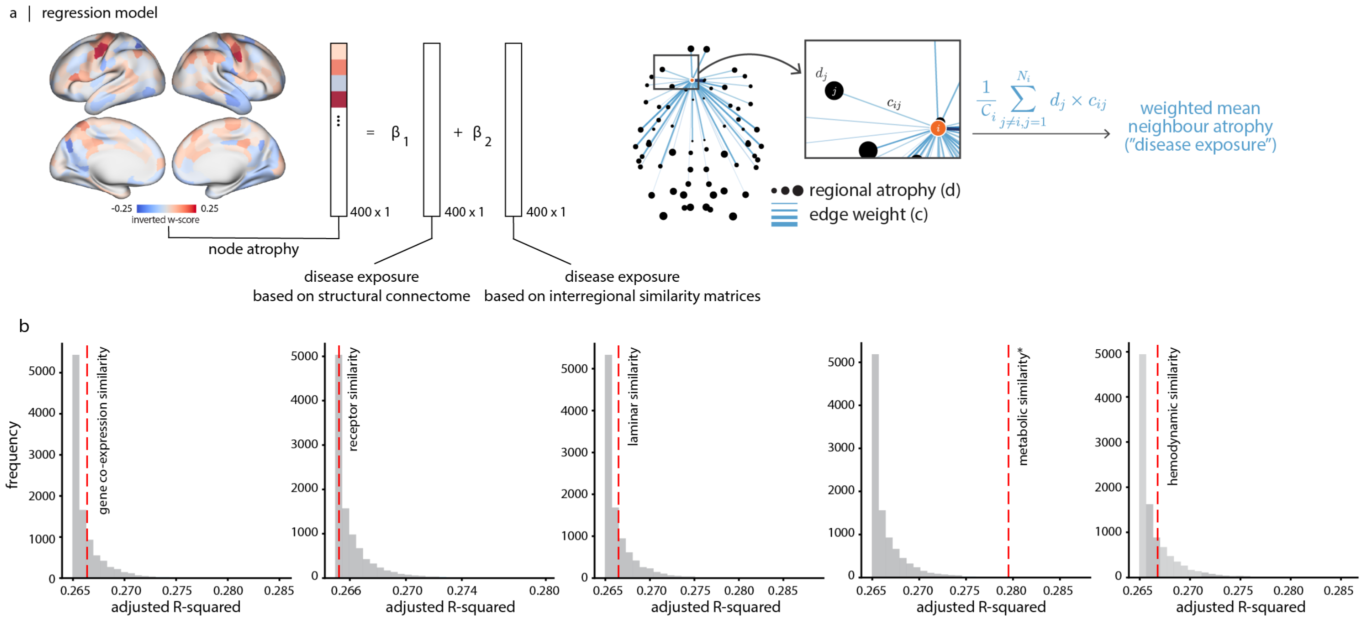

Figure S5. **Joint contribution of structural connectome and metabolic inter-regional similarity matrices in shaping ALS cortical atrophy |** (a) We build linear regression models to predict ALS cortical atrophy using two variables: (1) weighted mean neighbour atrophy, where the weights are derived from the structural connectome, and (2) weighted mean neighbour atrophy, where the weights come from the interregional similarity matrices. (b) We assess whether adding the second regressor (weighted mean neighbour atrophy based on a specific interregional similarity matrix) improves the model fit (adjusted R^2), compared to adding a regressor with the same spatial organization. Here we spun the inter-regional similarity-based weighted neighbour atrophy map, while keeping the weighted neighbour atrophy map coming from the structural connectome intact and recalculated the adjusted R^2 values ($n_{spin} = 10,000$). The histograms show the distribution of adjusted R^2 values when including the spunned regressors, the actual adjusted R^2 values are indicated by red lines. The metabolic similarity matrix led to an improvement in the adjusted R^2 value (increase in adjusted R^2 from 0.266 to 0.280, FDR corrected; $p_{\text{spin}} = 1.40 \times 10^{-2}$).

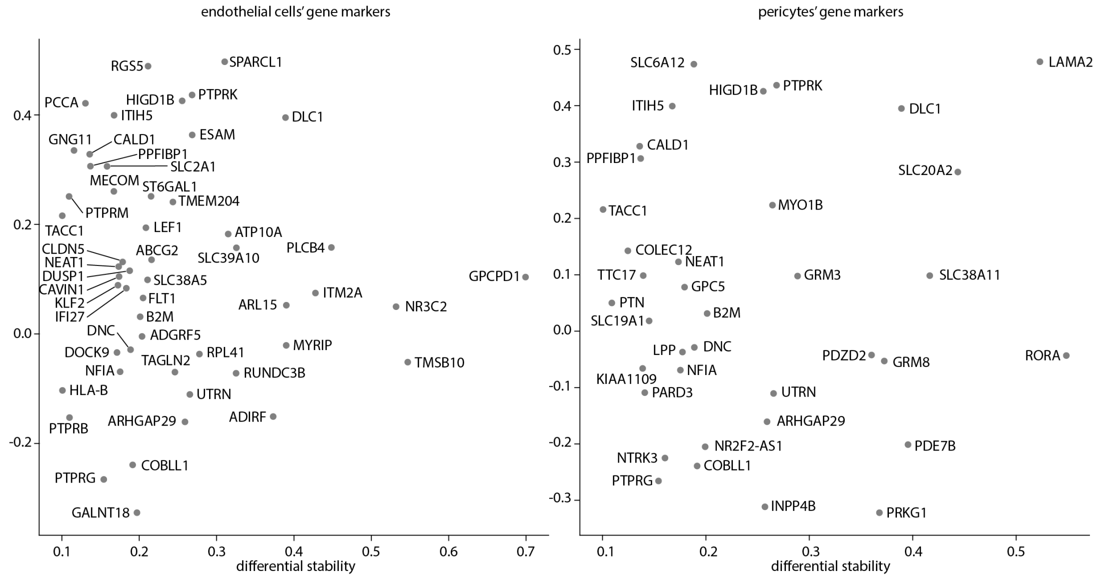

Figure S6. **Gene markers for endothelial cells and pericytes |** The y-axis shows the Spearman correlation coefficient between the gene maps and the epicenter map shown in Fig. 2c. The x-axis represents the differential stability values of the genes. The differential stability reflects the consistency of a gene across the six human brains provided by the Allen Human Brain Atlas, with higher differential stability indicating greater reliability. The gene markers are developed by Lake et al. [9].

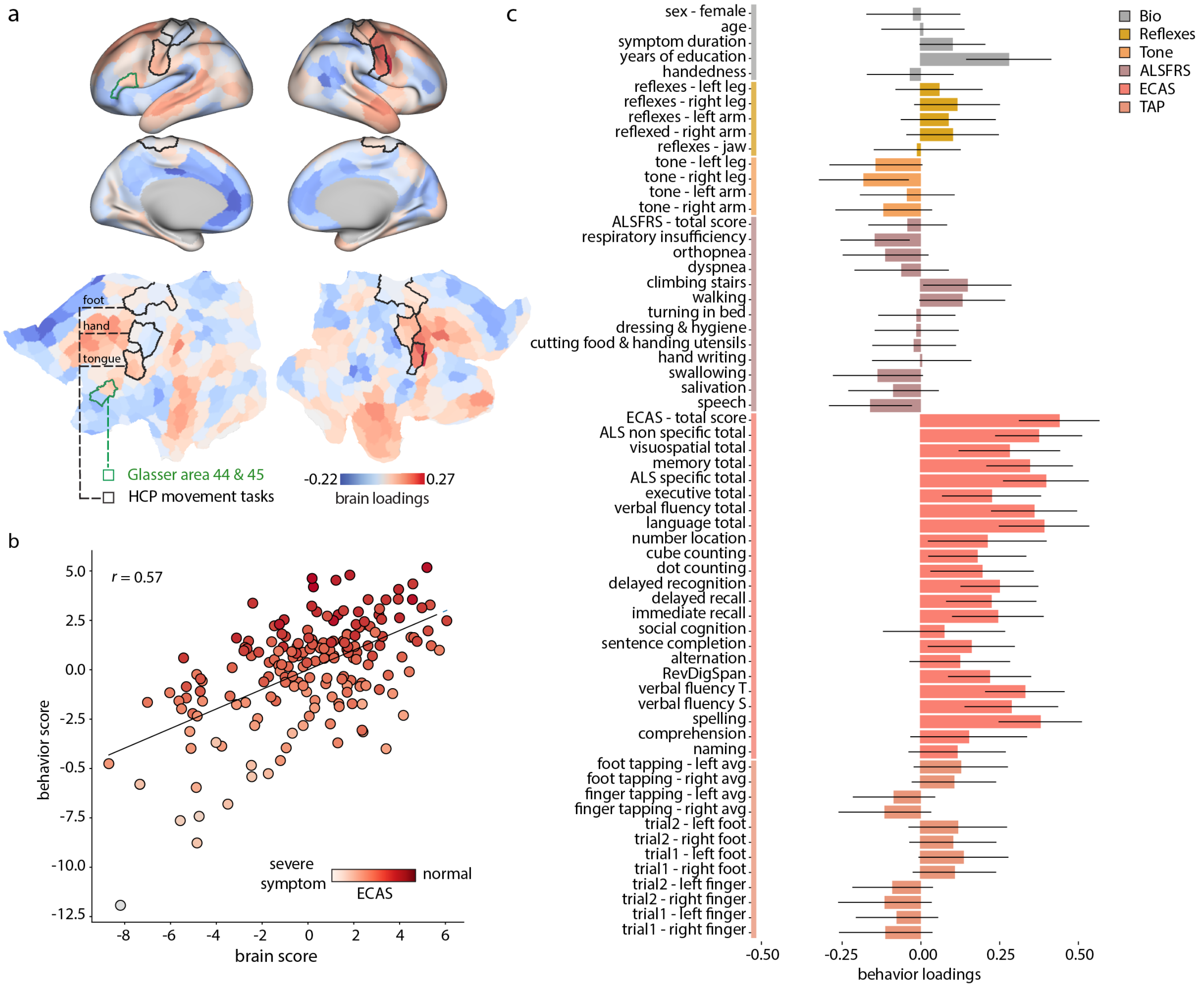

Figure S7. **Second latent variable from a PLS analysis to relate individual epicenter maps with clinical and behavioral measures |** (a) Brain loadings are shown on the fs-LR inflated and flat cortical surfaces. Regions demarcated by the black border are those with the greatest effect sizes (parcels in which over 50% of the vertices have a Cohen's d effect size exceeding 1) in the group-average activation map from S1200 Human Connectome Project package for the movement task contrasts [10]. Regions demarcated by green borders showcase areas 44 and 45 from the Glasser parcellation [11]. These regions, specifically in the left hemisphere [12], correspond to the Broca's area [13]. (b) The scatter plot visualizes the individual participants' brain scores versus behavioral PLS scores (Pearson correlation coefficient, $r = 0.57$; Spearman correlation coefficient, $r = 0.50$); each participant's score is colored based on the ECAS total score. (c) The bar plot visualizes the behavioral/clinical measures' loadings. The contribution (effect size) of individual variables is assessed by bootstrap resampling ($1,000$ repetitions).

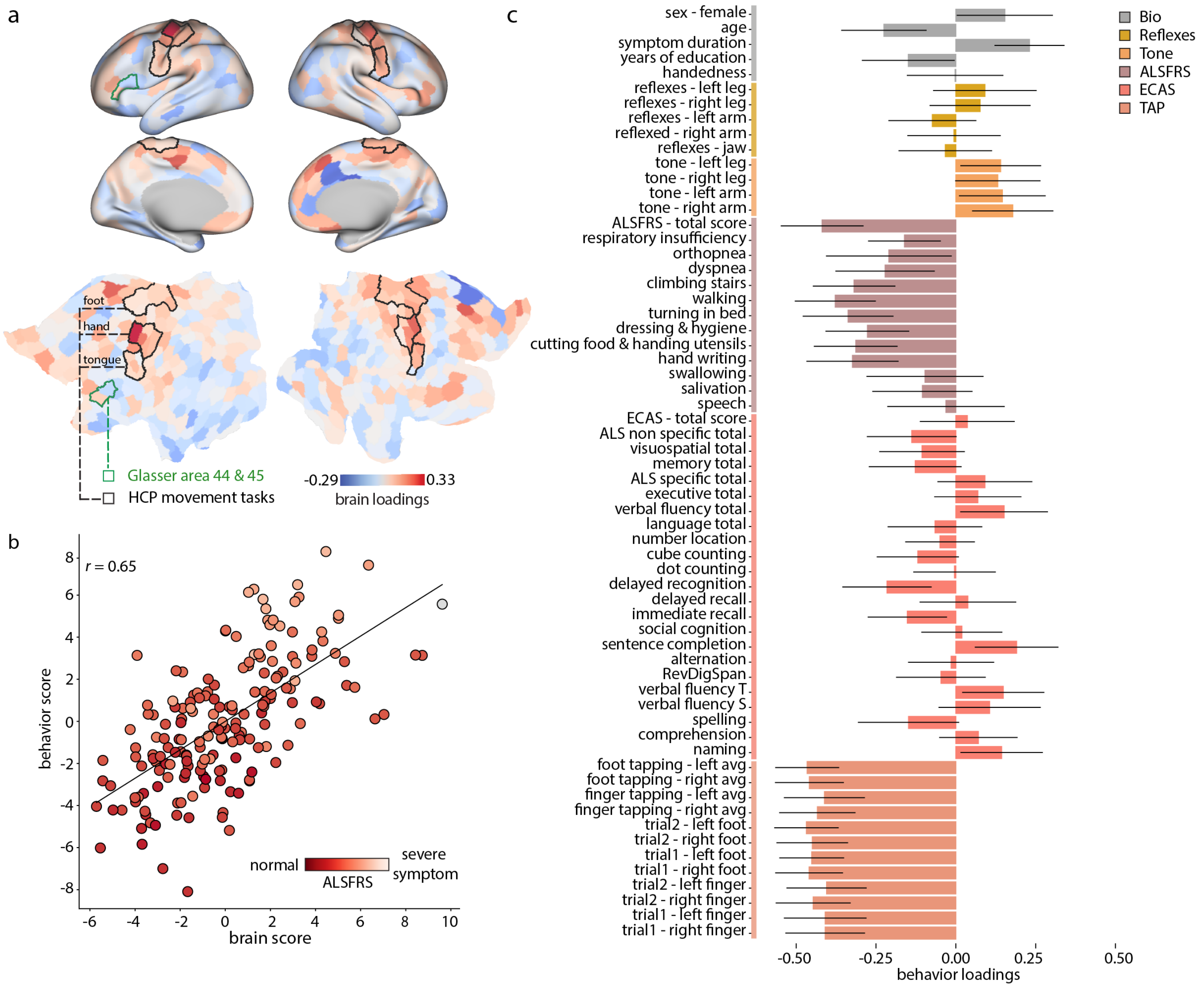

Figure S8. **First latent variable from a PLS analysis to relate individual atrophy maps with clinical and behavioral measures | ** The first latent variable in this case captures 20.22% of variance in the data ($p_{\text{permutation}} = 1.99 \times 10^{-3}$). (a) Brain loadings are shown on the fs-LR inflated and flat cortical surfaces. Regions demarcated by the black border are those with the greatest effect sizes (parcels in which over 50% of the vertices have a Cohen's d effect size exceeding 1) in the group-average activation map from S1200 Human Connectome Project package for the movement task contrasts [10]. Regions demarcated by green borders showcase areas 44 and 45 from the Glasser parcellation [11]. These regions, specifically in the left hemisphere [12], correspond to the Broca's area [13]. (b) The scatter plot visualizes the individual participants' brain scores versus behavioral PLS scores (Pearson correlation coefficient, $r = 0.65$; Spearman correlation coefficient, $r = 0.67$); each participant's score is colored based on the ALSFRS total score. (c) The bar plot visualizes the behavioral/clinical measures' loadings. The contribution (effect size) of individual variables is assessed by bootstrap resampling (1,000 repetitions).

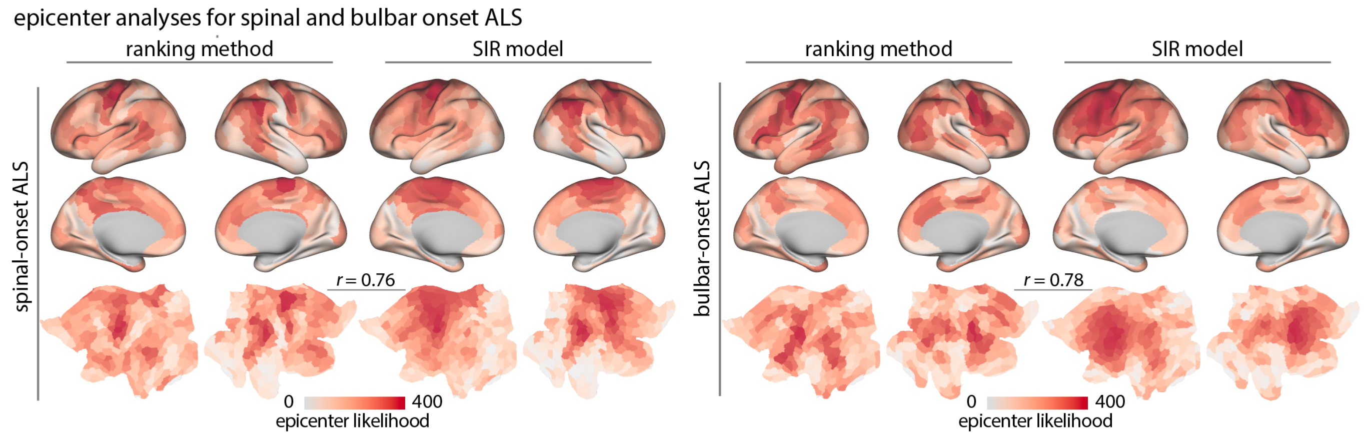

Figure S9. **Atrophy epicenters in spinal- and bulbar-onset ALS |** Epicenter likelihood maps for spinal-onset ALS (left) and bulbar-onset ALS (right). Maps are obtained for each subtype using two methodologies: ranking approach and the agent-based SIR modeling approach. The maps derived by the two methods are correlated with each other, for both subtypes (Pearson correlation coefficient; $r_{\text{spinal}} = 0.76$, $r_{\text{bulbar}} = 0.78$, $p_{\text{spin}} = 9.99 \times 10^{-4}$).

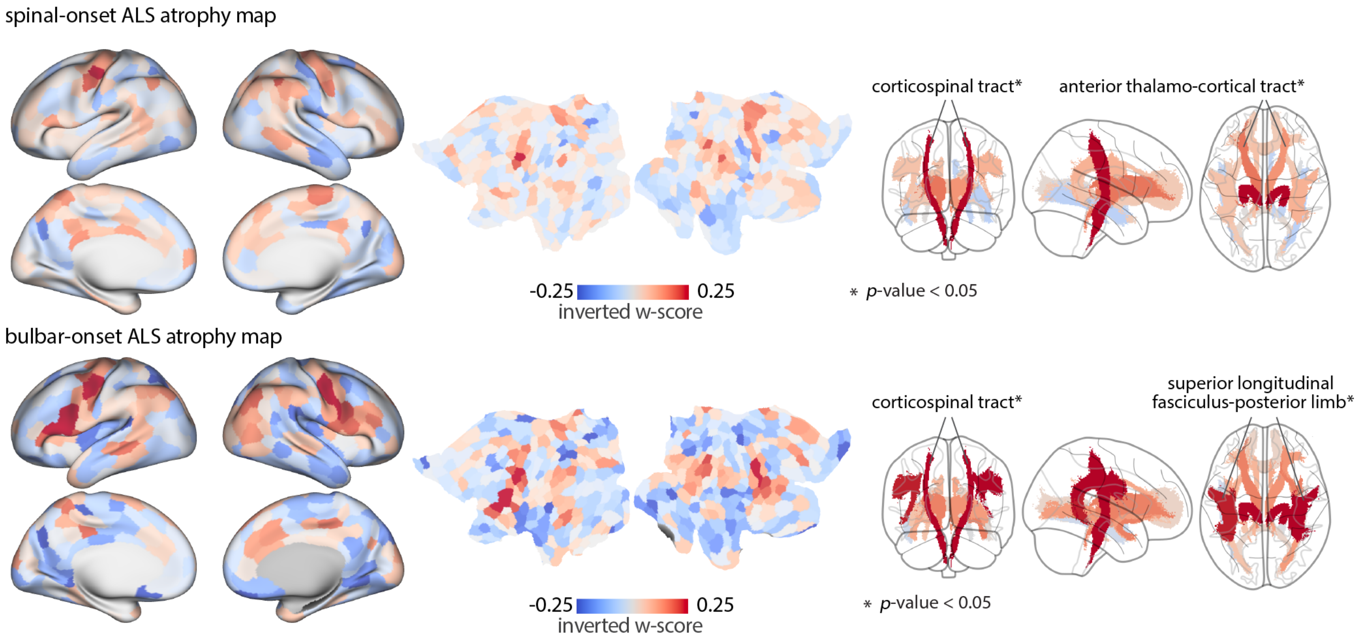

Figure S10. **Map of ALS atrophy stratified by type of disease onset |** Measuring the atrophy within atlas-based defined tracts [14, 15] showed that while corticospinal tract is involved in both spinal-(FDR corrected; left: $p = 1.45 \times 10^{-9}$, t-statistic $= 7.50$; right: $p = 7.72 \times 10^{-10}$, t-statistic $= 7.74$) and bulbar-onset ALS (FDR corrected; left: $p = 1.72 \times 10^{-3}$, t-statistic $= 5.26$; right: $p = 5.52 \times 10^{-4}$, t-statistic $= 4.40$). For patients with the spinal-onset of the disease, the anterior thalamic region is also significantly atrophied (FDR corrected; left: $p = 4.31 \times 10^{-5}$, t-statistic $= 5.34$; right: $p = 9.62 \times 10^{-4}$, t-statistic $= 4.55$). The involvement of anterior thalamic radiation in ALS has been reported in the cited references [16, 17]. In patients with the bulbar-onset of the disease, significant atrophy is also observed in the superior longitudinal fasciculus tract (FDR corrected; left: $p = 1.72 \times 10^{-3}$, t-statistic $= 4.88$; right: $p = 5.34 \times 10^{-3}$, t-statistic $= 4.40$). This tract is known to play role in speech [18] and language functions [19]. Decrease in fractional anisotropy of the superior longitudinal fasciculus tract in bulbar-onset ALS patients has been reported in previous studies [20, 21].

Study	Site	Control			ALS		
		**Age (years)**	**%Female**	**#Participants**	**Age (years)**	**%Female**	**#Participants**
CALSNIC1	University of Calgary	54.30	60.00	10	58.00	66.67	9
CALSNIC1	University of Alberta	58.35	45.00	20	57.50	45.00	20
CALSNIC1	McGill University	53.86	28.57	7	62.38	15.38	13
CALSNIC1	University of Toronto	46.20	70.00	10	52.80	33.33	15
CALSNIC1	University of British Columbia	52.88	75.00	8	58.57	28.57	7
CALSNIC2	University of Calgary	59.30	50.00	10	55.50	33.33	6
CALSNIC2	University of Alberta	55.69	57.78	45	59.37	46.51	43
CALSNIC2	University of Miami	51.55	54.55	11	63.86	28.57	7
CALSNIC2	McGill University	53.24	35.29	17	59.06	17.65	17
CALSNIC2	Université Laval	62.65	76.47	17	62.29	28.57	21
CALSNIC2	University of Toronto	53.17	50.00	18	64.04	40.74	27
CALSNIC2	University of Utah	65.50	50.00	2	55.43	28.57	7

TABLE S1. **Demographics summary for ALS patients and controls stratified by study phase and imaging site**. In the column named “#Participants”, “#” stands for “number”.

Project	CALSNIC-1				CALSNIC-2			
	**University of Alberta**	**University of British Columbia**	**University of Calgary University of Toronto**	**McGill University**	**University of Alberta McGill University University of Toronto University of Utah**	**University of Calgary**	**University of Miami**	**Université Laval**
**Scanner model**	Siemens 3T Prisma	Philips 3T Intera	GE 3T Discovery MR 750	Siemens 3T TIM Trio	Siemens 3T Prisma[1]	GE 3T Discovery MR 750	Siemens 3T TIM Trio	Philips 3T Achieva TX
**Software version**	syngo MR E11	3.2.3.1	DV25.0EB1442.a	syngo MR B17	DV25.0R021549.b	syngo MR B17	syngo MR B17	5.3.0.3
**Coil, channels**	20	8	12[2]	32	64/20[3]	32/8[3]	12	8
**Acquisition type**	2D	2D	2D	2D	3D	3D	3D	3D
**Acquisition orientation**	Axial	Axial	Axial	Axial	Sagittal	Sagittal	Sagittal	Sagittal
**Repetition time, ms**	2300	7.9	min (7.4)	2300	1700	8.1	1800	Shortest (7.1)
**Echo time, ms**	3.43	3.5 min	min(3.1)	3.43	2.21	Min full (3.2)	2.13	Short (3.4)
**Inversion time, ms**	900	950	400	900	880	400	900	950
**Flip angle, degrees**	9	8	11	9	10	16	10	10
**Field of view, mm**	256 x 256	240 x 240	256 x 256	256 x 256	232 x 256	256 x 256	256 x 256	256 x 256
**Matrix dimensions, pixels**	256 x 256	240 x 240	256 x 256	256 x 256	232 x 256	224 x 256	230 x 256 (272 x 320)	256 x 256
**Voxel dimensions, mm**	1 x 1 x 1	1 x 1 x 1	1 x 1 x 1	1 x 1 x 1	1 x 1 x 1	1 x 1 x 15	1 x 1 x 15	1 x 1 x 1
**Slices, n**	176	150	176	176	176	176	176	176
**Acquisition time, mins**	05:30	06:08	04:30	05:30	03:37	04:16	04:10	04:08

TABLE S2. **Scanner and imaging sequence specifications across different imaging sites**

[1] Different scanner model used at McGill University: Siemens 3T Prisma Fit

[2] Different coil used at the different centers: University of Calgary, 12 ch; University of Toronto, 8 ch; Western University, 32 ch.

[3] For CALSNIC-2 at centers where two coils were available, parameters are shown for the preferred coil (64 channel on Prisma, 32 channel on MR750)

Notation	Name	Expression or Value	Explanation
Δt	Time step	$\Delta t = 0.02$	Time increments in the simulations
α_i	synthesis rate in region i	equal for all regions ($= 0.5$)	Probability that a new normal agent gets synthesized in each voxel of region i per unit time
β_i	clearance rate in region i	equal for all regions ($= 0.5$)	Probability that an existing agent (either normal or misfolded) in region i gets cleared per unit time
ρ_i	Probability of remaining in region i	$\rho_i = 0.99$ for all i	Agents in region i have equal probability of remaining or exiting per unit time
k_1	Weight of atrophy accrual due to accumulation of misfolded agents	$k_1 = 0.5$	Contribution of native misfolded agents to total atrophy growth
k_2	Weight of atrophy accrual due to deafferentation	$k_2 = 0.5$	Contribution of deafferentation to total atrophy growth
w_{ij}	Connection strength between regions i and j	Streamline density	Strength of the connection between regions based on streamline density
l_{ij}	Connection length between regions i and j	Euclidean length of streamline fibers	Physical distance between regions based on fiber tract length

TABLE S3. **Parameters of the agent-based SIR model |** For more information on the model equations, refer to the *Supplementary Information: The S.I.R model* section.

	Variable Name	Description
1	Sex	Female: 1, Male: 0
2	Age	Participant age at the date of screening
3	Symptom duration	Symptom duration in months from time of onset to date of baseline visit
4	Years of education	Years of education beginning at grade 1
5	Handedness	Hand preference
6	Reflexes – left leg	0: Normal, decreased or absent; 1: Increased; 2: Clonus
7	Reflexes – right leg	0: Normal, decreased or absent; 1: Increased; 2: Clonus
8	Reflexes – left arm	0: Normal, decreased or absent; 1: Increased; 2: Clonus
9	Reflexes – right arm	0: Normal, decreased or absent; 1: Increased; 2: Clonus
10	Reflexes – jaw	0: Normal, decreased or absent; 1: Increased; 2: Clonus
11	Tone – left leg	0: No increase in tone; 1: Increase in tone
12	Tone – right leg	0: No increase in tone; 1: Increase in tone
13	Tone – left arm	0: No increase in tone; 1: Increase in tone
14	Tone – right arm	0: No increase in tone; 1: Increase in tone
15	ALSFRS Total Score	Total ALSFRS score out of 48
16	ALSFRS Respiratory Insufficiency	4: None, 3: Intermittent use of bilevel positive airway pressure (BiPAP), 2: Continuous nocturnal BiPAP, 1: Continuous use during night and day, 0: Invasive mechanical ventilation
17	ALSFRS Orthopnea	4: None, 3: Some difficulty sleeping at night due to shortness of breath, 2: Needs extra pillows in order to sleep, 1: Can only sleep sitting up, 0: Unable to sleep
18	ALSFRS Dyspnea	4: None, 3: Occurs when walking, 2: Occurs with one or more of the following activities: eating, bathing, dressing, 1: Occurs at rest, difficulty breathing either siting or lying, 0: Significant difficulty, considering using mechanical support
19	ALSFRS Climbing stairs	4: Normal, 3: Slow, 2: Mild unsteadiness or fatigue, 1: Needs assistance, 0: Cannot do
20	ALSFRS Walking	4: Normal, 3: Early ambulation difficulties, 2: Walks with assistance, 1: Non-ambulatory functional movement, 0: No purposeful leg movement
21	ALSFRS Turning in bed	4: Normal, 3: Somewhat slow and clumsy but no help needed , 2: Can turn alone with difficulty, 1: Can initiate but not turn alone, 0: Helpless
22	ALSFRS Dressing & hygiene	4: Normal function, 3: Independent and complete self-care with effort or decreased efficiency, 2: Intermittent assistance, or substitute methods, 1: Needs attendant for self-care, 0: Total dependence
23	ALSFRS Cutting food & handling utensils	4: Normal, 3: Slow but no help needed, 2: Some help needed, 1: Needs food cut, 0: Needs to be fed
24	ALSFRS Handwriting	4: Normal, 3: Slow or sloppy but legible, 2: Not all words legible, 1: Able to grip pen but can't write, 0: Unable to grip pen
25	ALSFRS Swallowing	4: Normal, 3: Early eating problems, 2: Dietary consistency changes, 1: Needs supplemental feeding, 0: NPO (parenteral or enteral feeding)
26	ALSFRS Salivation	4: Normal, 3: Slight excess of saliva in mouth and may have night-time drooling, 2: Moderate excess with minimal drooling, 1: Marked excess of saliva with some drooling, 0: Needs constant tissue or handkerchief
27	ALSFRS Speech	4: Normal speech processes, 3: Detectable speech disturbance, 2: Intelligible with repeating, 1: Speech combined with non-vocal communication, 0: Loss of useful speech
28	ECAS Total Score	Total ECAS score (ALS specific and non-specific domains) out of 136
29	ECAS ALS Non Specific_Total	ALS non-specific total score (Memory, Visuospatial) out of 36
30	ECAS Visuospatial Total	Visuospatial domain total score (Dot counting, Cube counting, Number location) out of 12
31	ECAS Memory Total	Memory domain total score (Immediate recall, Delayed retention, Delayed recognition) out of 24
32	ECAS ALS Specific Total	ALS specific domains total score (Language, Verbal Fluency, Executive) out of 100
33	ECAS Executive Total	Executive domain total score (Reverse digit span, Alternation, Sentence completion, Social cognition) out of 48
34	ECAS Verbal Fluency Total	Verbal Fluency domain total score (Fluency Letter S, Fluency Letter T) out of 24

Continued on next page

Continued from previous page

35	ECAS Language Total	Language domain total score (Naming, Comprehension, Spelling) out of 28
36	ECAS Number Location	VISUOSPATIAL – Number Location score out of 4
37	ECAS Cube Counting	VISUOSPATIAL – Cube Counting score out of 4
38	ECAS Dot Counting	VISUOSPATIAL – Dot Counting score out of 4
39	ECAS Delayed Recognition	MEMORY – Delayed Recognition score out of 4
40	ECAS Delayed Recall	MEMORY – Delayed Recall converted score out of 10
41	ECAS Immediate Recall	MEMORY – Immediate Recall score out of 10
42	ECAS Social Cognition	EXECUTIVE – Social Cognition score out of 12
43	ECAS Sentence Completion	EXECUTIVE – Sentence Completion score out of 12
44	ECAS Alternation	EXECUTIVE – Alternation score out of 12
45	ECAS RevDigSpan	EXECUTIVE – Reverse Digit Span score out of 12
46	ECAS VerbFluencyT	FLUENCY – Fluency Letter T score out of 12
47	ECAS VerbFluencyS	FLUENCY – Fluency Letter S score out of 12
48	ECAS Spelling	FLUENCY – Spelling score out of 12
49	ECAS Comprehension	LANGUAGE – Comprehension score out of 8
50	ECAS Naming	LANGUAGE – Naming score out of 8
51	Foot tapping – left avg	Average of Trial 1 and 2 for Left Foot
52	Foot tapping – right avg	Average of Trial 1 and 2 for Right Foot
53	Finger tapping – left avg	Average of Trial 1 and 2 for Left Index Finger
54	Finger tapping – right avg	Average of Trial 1 and 2 for Right Index Finger
55	Foot tapping – Trial 2 – left foot	Score of Trial 2 for Left Foot (number of taps in 10 second period)
56	Foot tapping – Trial 2 – right foot	Score of Trial 2 for Right Foot (number of taps in 10 second period)
57	Foot tapping – Trial 1 – left foot	Score of Trial 1 for Left Foot (number of taps in 10 second period)
58	Foot tapping – Trial 1 – right foot	Score of Trial 1 for Right Foot (number of taps in 10 second period)
59	Finger tapping – Trial 2 – left finger	Score of Trial 2 for Left Index Finger (number of taps in 10 second period)
60	Finger tapping – Trial 2 – right finger	Score of Trial 2 for Right Index Finger (number of taps in 10 second period)
61	Finger tapping – Trial 1 – left finger	Score of Trial 1 for Left Index Finger (number of taps in 10 second period)
62	Finger tapping – Trial 1 – right finger	Score of Trial 1 for Right Index Finger (number of taps in 10 second period)

TABLE S4: **Details of measures used in the partial least square (PLS) analysis**

Region of onset	#Participants
lower_extremity	69
upper_extremity	64
upper_extremity & lower_extremity	7
bulbar	10
bulbar_speech	18
bulbar_speech & bulbar_swallowing	10
bulbar_speech & upper_extremity	2
bulbar & lower_extremity	1
bulbar & upper_extremity	1
bulbar_swallowing & upper_extremity	1
bulbar_speech & lower_extremity	1
bulbar_speech & upper_extremity & lower_extremity	1
upper_extremity & cognitive	1

TABLE S5. **Region of onset and corresponding ALS patient counts |** Rows highlighted in blue represent onset regions we refer to as "spinal-onset ALS" throughout the manuscript, and the ones highlighted in red represent onset regions we refer to as "bulbar-onset ALS" throughout the manuscript.

Receptor/ transporter	Neurotransmitter	Tracer	Measure	#Participants	Age (years)	References
D_1	dopamine	$[^{11}C]$SCH23390	BP_{ND}	13 (7)	33 ± 13	Kaller et al., 2017 [22]
D_2	dopamine	$[^{11}C]$FLB-457	BP_{ND}	37 (20)	48.4 ± 16.9	Smith et al., 2019 [23, 24]
D_2	dopamine	$[^{11}C]$FLB-457	BP_{ND}	55 (29)	32.5 ± 9.7	Sandiego et al., 2015 [23–27]
DAT*	dopamine	$[^{123}I]$-FP-CIT	SUVR	174 (65)	61 ± 11	Dukart et al., 2018 [28]
NET*	norepinephrine	$[^{11}C]$MRB	BP_{ND}	77 (27)	33.4 ± 9.2	Ding et al., 2010 [29–31]
5-HT_{1A}	serotonin	$[^{11}C]$WAY-100635	BP_{ND}	35 (17)	26.3 ± 5.2	Savli et al., 2012 [32]
5-HT_{1B}	serotonin	$[^{11}C]$P943	BP_{ND}	65 (16)	33.7 ± 9.7	Gallezot et al., 2010 [33–39]
5-HT_{1B}	serotonin	$[^{11}C]$P943	BP_{ND}	23 (8)	28.7 ± 7.0	Savli et al., 2012 [32]
5-HT_{2A}	serotonin	$[^{11}C]$Cimbi-36	B_{max}	29 (14)	22.6 ± 2.7	Beliveau et al., 2017 [40]
5-HT_4	serotonin	$[^{11}C]$SB207145	B_{max}	59 (18)	25.9 ± 5.3	Beliveau et al., 2017 [40]
5-HT_6	serotonin	$[^{11}C]$GSK215083	BP_{ND}	30 (0)	36.6 ± 9.0	Radhakrishnan et al., 2018 [41, 42]
5-HTT*	serotonin	$[^{11}C]$DASB	B_{max}	100 (71)	25.1 ± 5.8	Beliveau et al., 2017 [40]
$\alpha_4\beta_2$	acetylcholine	$[^{18}F]$flubatine	V_T	30 (10)	33.5 ± 10.7	Hillmer et al., 2016 [43, 44]
M_1	acetylcholine	$[^{11}C]$LSN3172176	BP_{ND}	24 (11)	40.5 ± 11.7	Naganawa et al., 2021 [45]
VAChT*	acetylcholine	$[^{18}F]$FEOBV	SUVR	4 (1)	37 ± 10.2	PI: L Tuominen & S Guimond
VAChT*	acetylcholine	$[^{18}F]$FEOBV	SUVR	18 (13)	66.8 ± 6.8	Aghourian et al., 2017 [46]
VAChT*	acetylcholine	$[^{18}F]$FEOBV	SUVR	5 (1)	68.3 ± 3.1	Bedard et al., 2019 [47]
VAChT*	acetylcholine	$[^{18}F]$FEOBV	SUVR	3 (3)	66.6 ± 0.94	PI: TW Schmitz & RN Spreng
NMDA	glutamate	$[^{18}F]$GE-179	V_T	29 (8)	40.9 ± 12.7	Galovic et al., 2021 [48–50]
$mGluR_5$	glutamate	$[^{11}C]$ABP688	BP_{ND}	73 (48)	19.9 ± 3.04	Smart et al., 2019 [51]
$mGluR_5$	glutamate	$[^{11}C]$ABP688	BP_{ND}	22 (10)	67.9 ± 9.6	PI: P Rosa-Neto & E Kobayashi
$mGluR_5$	glutamate	$[^{11}C]$ABP688	BP_{ND}	28 (13)	33.1 ± 11.2	DuBois et al., 2016 [52]
$GABA_{A/BZ}$	GABA	$[^{11}C]$flumazenil	B_{max}	16 (9)	26.6 ± 8	Nørgaard et al., 2021 [53]
H_3	histamine	$[^{11}C]$GSK189254	V_T	8 (1)	31.7 ± 9.0	Gallezot et al., 2017 [54]
CB_1	cannabinoid	$[^{11}C]$OMAR	V_T	77 (28)	30.0 ± 8.9	Normandin et al., 2015 [55–58]
MOR	opioid	$[^{11}C]$carfentanil	BP_{ND}	204 (72)	32.3 ± 10.8	Kantonen et al., 2020 [59]

TABLE S6. **Neurotransmitter receptors and transporters used to build the receptor interregional similarity matrix |** BP_{ND} = non-displaceable binding potential; V_T = tracer distribution volume; B_{max} = density (pmol/ml) converted from binding potential (5-HT) or distributional volume (GABA) using autoradiography-derived densities; SUVR = standard uptake value ratio. Values in parentheses (under #Participants) indicate number of females. Neurotransmitter receptor maps without citations refer to previously unpublished data. In those cases, contact information for the study principal investigator is provided in the Table. Asterisks indicate transporters.

Variable Name	#Participants with missing data
Symptom duration	6
Years of education	6
Handedness	6
Reflexes – right leg	15
Reflexes – left leg	16
Reflexes – arm	16
Reflexes – jaw	19
Tone – leg	17
Tone – arm	16
ALSFRS measures	5
ECAS measures	12
Foot tapping	12
Finger tapping	11

TABLE S7. **Number of ALS participants with missing values for the PLS analysis |** To conduct the PLS analysis, missing values in the data are handled by imputing the median value of the corresponding variable. This table shows the number of participants with missing values for each measure. Total number of ALS participants included in the PLS analysis is equal to 184.

Variable Name	#Spinal-onset participants with missing data	#Bulbar-onset participants with missing data
Reflexes – right leg	3	8
Reflexes – left leg	3	9
Reflexes – arm	3	9
Reflexes – jaw	4	11
Tone – leg	3	10
Tone – arm	3	9
ALSFRS measures	2	2
ECAS measures	4	12
Foot tapping	4	13
Finger tapping	4	12

TABLE S8. **Number of ALS participants with missing values stratified by ALS region of onset, related to Fig.** 6**b |** To compare the behavioral/clinical measures between the subtypes (spinal and bulbar onset patients), missing values in the data are removed prior to conducting the statistical tests. The table presents the number of participants with missing data for each measure. In total, 38 participants are classified as bulbar-onset ALS and 140 participants are classified as spinal-onset ALS.

[1] Ying-Qiu Zheng, Yu Zhang, Yvonne Yau, Yashar Zeighami, Kevin Larcher, Bratislav Misic, and Alain Dagher. Local vulnerability and global connectivity jointly shape neurodegenerative disease propagation. *PLoS biology*, 17(11):e3000495, 2019.

[2] Alaa Abdelgawad, Shady Rahayel, Ying-Qiu Zheng, Christina Tremblay, Andrew Vo, Bratislav Misic, and Alain Dagher. Predicting longitudinal brain atrophy in parkinson's disease using a susceptible-infected-removed agent-based model. *Network Neuroscience*, 7(3):906–925, 2023.

[3] Shady Rahayel, Christina Tremblay, Andrew Vo, Ying Qiu Zheng, Stéphane Lehéricy, Isabelle Arnulf, Marie Vidailhet, Jean Christophe Corvol, Jean François Gagnon, et al. Brain atrophy in prodromal synucleinopathy is shaped by structural connectivity and gene expression. *Brain*, 145(9):3162–3178, 2022.

[4] Golia Shafiei, Vincent Bazinet, Mahsa Dadar, Ana L Manera, D Louis Collins, Alain Dagher, Barbara Borroni, Raquel Sanchez-Valle, Fermin Moreno, Robert Laforce Jr, et al. Network structure and transcriptomic vulnerability shape atrophy in frontotemporal dementia. *Brain*, 146(1):321–336, 2023.

[5] Constantin Economo et al. Die cytoarchitektonik der hirnrinde des erwachsenen menschen. *Arch NeurPsyche*, 1925.

[6] Constantin Freiherr von Economo, Georg N Koskinas, and Lazaros C Triarhou. *Atlas of cytoarchitectonics of the adult human cerebral cortex*, volume 10. Karger Basel, 2008.

[7] Lianne H Scholtens, Marcel A de Reus, Siemon C de Lange, Ruben Schmidt, and Martijn P van den Heuvel. An mri von economo–koskinas atlas. *NeuroImage*, 170:249–256, 2018.

[8] Alexander Schaefer, Ru Kong, Evan M Gordon, Timothy O Laumann, Xi-Nian Zuo, Avram J Holmes, Simon B Eickhoff, and BT Thomas Yeo. Local-global parcellation of the human cerebral cortex from intrinsic functional connectivity mri. *Cerebral cortex*, 28(9):3095–3114, 2018.

[9] Blue B Lake, Song Chen, Brandon C Sos, Jean Fan, Gwendolyn E Kaeser, Yun C Yung, Thu E Duong, Derek Gao, Jerold Chun, Peter V Kharchenko, et al. Integrative single-cell analysis of transcriptional and epigenetic states in the human adult brain. *Nature biotechnology*, 36(1):70–80, 2018.

[10] Deanna M Barch, Gregory C Burgess, Michael P Harms, Steven E Petersen, Bradley L Schlaggar, Maurizio Corbetta, Matthew F Glasser, Sandra Curtiss, Sachin Dixit, Cindy Feldt, et al. Function in the human connectome: task-fmri and individual differences in behavior. *Neuroimage*, 80:169–189, 2013.

[11] Matthew F Glasser, Timothy S Coalson, Emma C Robinson, Carl D Hacker, John Harwell, Essa Yacoub, Kamil Ugurbil, Jesper Andersson, Christian F Beckmann, Mark Jenkinson, et al. A multi-modal parcellation of human cerebral cortex. *Nature*, 536(7615):171–178, 2016.

[12] Reza Rajimehr, Arsalan Firoozi, Hossein Rafipoor, Nooshin Abbasi, and John Duncan. Complementary hemispheric lateralization of language and social processing in the human brain. *Cell Reports*, 41(6), 2022.

[13] S Catrin Blank, Sophie K Scott, Kevin Murphy, Elizabeth Warburton, and Richard JS Wise. Speech production: Wernicke, broca and beyond. *Brain*, 125(8):1829–1838, 2002.

[14] Setsu Wakana, Arvind Caprihan, Martina M Panzenboeck, James H Fallon, Michele Perry, Randy L Gollub, Kegang Hua, Jiangyang Zhang, Hangyi Jiang, Prachi Dubey, et al. Reproducibility of quantitative tractography methods applied to cerebral white matter. *Neuroimage*, 36(3):630–644, 2007.

[15] Kegang Hua, Jiangyang Zhang, Setsu Wakana, Hangyi Jiang, Xin Li, Daniel S Reich, Peter A Calabresi, James J Pekar, Peter CM van Zijl, and Susumu Mori. Tract probability maps in stereotaxic spaces: analyses of white matter anatomy and tract-specific quantification. *Neuroimage*, 39(1):336–347, 2008.

[16] Xiao-Qiang Du, Tian-Xiu Zou, Nao-Xin Huang, Zhang-Yu Zou, Yun-Jing Xue, and Hua-Jun Chen. Brain white matter abnormalities and correlation with severity in amyotrophic lateral sclerosis: An atlas-based diffusion tensor imaging study. *Journal of the Neurological Sciences*, 405:116438, 2019.

[17] Amutha Bharathi Mohan, Subathra Adithan, Sunil Narayan, Nagarajan Krishnan, and Donna Mathews. Evaluation of white matter tracts fractional anisotropy using tract-based spatial statistics and its correlation with amyotrophic lateral sclerosis functional rating scale score in patients with motor neuron disease. *Indian Journal of Radiology and Imaging*, 31(02):297–303, 2021.

[18] François Vassal, Fabien Schneider, Claire Boutet, Betty Jean, Anna Sontheimer, and Jean-Jacques Lemaire. Combined dti tractography and functional mri study of the language connectome in healthy volunteers: extensive mapping of white matter fascicles and cortical activations. *PloS one*, 11(3):e0152614, 2016.

[19] Riho Nakajima, Masashi Kinoshita, Harumichi Shinohara, and Mitsutoshi Nakada. The superior longitudinal fascicle: reconsidering the fronto-parietal neural network based on anatomy and function. *Brain imaging and behavior*, 14:2817–2830, 2020.

[20] Florian Borsodi, Valeriu Culea, Christian Langkammer, Michael Khalil, Lukas Pirpamer, Stefan Quasthoff, Christian Enzinger, Reinhold Schmidt, Franz Fazekas, and Stefan Ropele. Multimodal assessment of white matter tracts in amyotrophic lateral sclerosis. *PloS one*, 12(6):e0178371, 2017.

[21] Robert Steinbach, Tino Prell, Nayana Gaur, Annekathrin Roediger, Christian Gaser, Thomas E Mayer, Otto W Witte, and Julian Grosskreutz. Patterns of grey and white matter changes differ between bulbar and limb onset amyotrophic lateral sclerosis. *NeuroImage: Clinical*, 30:102674, 2021.

[22] Simon Kaller, Michael Rullmann, Marianne Patt, Georg-Alexander Becker, Julia Luthardt, Johanna Girbardt, Philipp M Meyer, Peter Werner, Henryk Barthel, Anke Bresch, et al. Test–retest measurements of dopamine d 1-type receptors using simultaneous pet/mri imaging. *European journal of nuclear medicine and molecular imaging*, 44(6):1025–1032, 2017.

[23] Christopher T Smith, Jennifer L Crawford, Linh C Dang, Kendra L Seaman, M Danica San Juan, Aishwarya Vijay, Daniel T Katz, David Matuskey, Ronald L Cowan, Evan D Morris, et al. Partial-volume correction increases estimated dopamine d2-like receptor binding potential and reduces adult age differences. *Journal of Cerebral Blood*

Flow & Metabolism, 39(5):822–833, 2019.

[24] Christine M Sandiego, Jean-Dominique Gallezot, Keunpoong Lim, Jim Ropchan, Shu-fei Lin, Hong Gao, Evan D Morris, and Kelly P Cosgrove. Reference region modeling approaches for amphetamine challenge studies with [11c] flb 457 and pet. *Journal of Cerebral Blood Flow & Metabolism*, 35(4):623–629, 2015.

[25] Yasmin Zakiniaeiz, Ansel T Hillmer, David Matuskey, Nabeel Nabulsi, Jim Ropchan, Carolyn M Mazure, Marina R Picciotto, Yiyun Huang, Sherry A McKee, Evan D Morris, et al. Sex differences in amphetamine-induced dopamine release in the dorsolateral prefrontal cortex of tobacco smokers. *Neuropsychopharmacology*, 44(13):2205–2211, 2019.

[26] Mark Slifstein, Elsmarieke Van De Giessen, Jared Van Snellenberg, Judy L Thompson, Rajesh Narendran, Roberto Gil, Elizabeth Hackett, Ragy Girgis, Najate Ojeil, Holly Moore, et al. Deficits in prefrontal cortical and extrastriatal dopamine release in schizophrenia: a positron emission tomographic functional magnetic resonance imaging study. *JAMA psychiatry*, 72(4):316–324, 2015.

[27] Christine M Sandiego, David Matuskey, Meaghan Lavery, Erin McGovern, Yiyun Huang, Nabeel Nabulsi, Jim Ropchan, Marina R Picciotto, Evan D Morris, Sherry A McKee, et al. The effect of treatment with guanfacine, an alpha2 adrenergic agonist, on dopaminergic tone in tobacco smokers: an [11c] flb457 pet study. *Neuropsychopharmacology*, 43(5):1052–1058, 2018.

[28] Juergen Dukart, Štefan Holiga, Christopher Chatham, Peter Hawkins, Anna Forsyth, Rebecca McMillan, Jim Myers, Anne R Lingford-Hughes, David J Nutt, Emilio Merlo-Pich, et al. Cerebral blood flow predicts differential neurotransmitter activity. *Scientific reports*, 8(1):1–11, 2018.

[29] Yu-Shin Ding, Tarun Singhal, Beata Planeta-Wilson, Jean-Dominique Gallezot, Nabeel Nabulsi, David Labaree, Jim Ropchan, Shannan Henry, Wendol Williams, Richard E Carson, et al. Pet imaging of the effects of age and cocaine on the norepinephrine transporter in the human brain using (s, s)-[11c] o-methylreboxetine and hrrt. *Synapse*, 64(1):30–38, 2010.

[30] Elizabeth Sanchez-Rangel, Jean-Dominique Gallezot, Catherine W Yeckel, Wai Lam, Renata Belfort-DeAguiar, Ming-Kai Chen, Richard E Carson, Robert Sherwin, and Janice J Hwang. Norepinephrine transporter availability in brown fat is reduced in obesity: a human pet study with [11c] mrb. *International Journal of Obesity*, 44(4):964–967, 2020.

[31] Renata Belfort-DeAguiar, Jean-Dominique Gallezot, Janice J Hwang, Ahmed Elshafie, Catherine W Yeckel, Owen Chan, Richard E Carson, Yu-Shin Ding, and Robert S Sherwin. Noradrenergic activity in the human brain: a mechanism supporting the defense against hypoglycemia. *The Journal of Clinical Endocrinology & Metabolism*, 103(6):2244–2252, 2018.

[32] Markus Savli, Andreas Bauer, Markus Mitterhauser, Yu-Shin Ding, Andreas Hahn, Tina Kroll, Alexander Neumeister, Daniela Haeusler, Johanna Ungersboeck, Shannan Henry, et al. Normative database of the serotonergic system in healthy subjects using multi-tracer pet. *Neuroimage*, 63(1):447–459, 2012.

[33] Jean-Dominique Gallezot, Nabeel Nabulsi, Alexander Neumeister, Beata Planeta-Wilson, Wendol A Williams, Tarun Singhal, Sunhee Kim, R Paul Maguire, Timothy McCarthy, J James Frost, et al. Kinetic modeling of the serotonin 5-ht1b receptor radioligand [11c] p943 in humans. *Journal of Cerebral Blood Flow & Metabolism*, 30(1):196–210, 2010.

[34] James W Murrough, Shannan Henry, Jian Hu, Jean-Dominique Gallezot, Beata Planeta-Wilson, John F Neumaier, and Alexander Neumeister. Reduced ventral striatal/ventral pallidal serotonin 1b receptor binding potential in major depressive disorder. *Psychopharmacology*, 213:547–553, 2011.

[35] James W Murrough, Christoph Czermak, Shannan Henry, Nabeel Nabulsi, Jean-Dominique Gallezot, Ralitza Gueorguieva, Beata Planeta-Wilson, John H Krystal, John F Neumaier, Yiyun Huang, et al. The effect of early trauma exposure on serotonin type 1b receptor expression revealed by reduced selective radioligand binding. *Archives of general psychiatry*, 68(9):892–900, 2011.

[36] David Matuskey, Zubin Bhagwagar, Beata Planeta, Brian Pittman, Jean-Dominique Gallezot, Jason Chen, Jane Wanyiri, Soheila Najafzadeh, Jim Ropchan, Paul Geha, et al. Reductions in brain 5-ht1b receptor availability in primarily cocaine-dependent humans. *Biological psychiatry*, 76(10):816–822, 2014.

[37] Christopher Pittenger, Thomas G Adams Jr, Jean-Dominique Gallezot, Michael J Crowley, Nabeel Nabulsi, James Ropchan, Hong Gao, Stephen A Kichuk, Ryan Simpson, Eileen Billingslea, et al. Ocd is associated with an altered association between sensorimotor gating and cortical and subcortical 5-ht1b receptor binding. *Journal of affective disorders*, 196:87–96, 2016.

[38] Aybala Saricicek, Jason Chen, Beata Planeta, Barbara Ruf, Kalyani Subramanyam, Kathleen Maloney, David Matuskey, David Labaree, Lorenz Deserno, Alexander Neumeister, et al. Test–retest reliability of the novel 5-ht 1b receptor pet radioligand [11 c] p943. *European journal of nuclear medicine and molecular imaging*, 42:468–477, 2015.

[39] Stephen R Baldassarri, Eunkyung Park, Sjoerd J Finnema, Beata Planeta, Nabeel Nabulsi, Soheila Najafzadeh, Jim Ropchan, Yiyun Huang, Jonas Hannestad, Kathleen Maloney, et al. Inverse changes in raphe and cortical 5-ht1b receptor availability after acute tryptophan depletion in healthy human subjects. *Synapse*, 74(10):e22159, 2020.

[40] Vincent Beliveau, Melanie Ganz, Ling Feng, Brice Ozenne, Liselotte Højgaard, Patrick M Fisher, Claus Svarer, Douglas N Greve, and Gitte M Knudsen. A high-resolution in vivo atlas of the human brain's serotonin system. *Journal of Neuroscience*, 37(1):120–128, 2017.

[41] Rajiv Radhakrishnan, Nabeel Nabulsi, Edward Gaiser, Jean-Dominique Gallezot, Shannan Henry, Beata Planeta, Shu-fei Lin, Jim Ropchan, Wendol Williams, Evan Morris, et al. Age-related change in 5-ht6 receptor availability in healthy male volunteers measured with 11c-gsk215083 pet. *Journal of Nuclear Medicine*, 59(9):1445–1450, 2018.

[42] Rajiv Radhakrishnan, David Matuskey, Nabeel Nabulsi, Edward Gaiser, Jean-Dominique Gallezot, Shannan Henry, Beata Planeta, Shu-fei Lin, Jim Ropchan, Yiyun Huang, et al. In vivo 5-ht6 and 5-ht2a receptor availability in antipsychotic treated schizophrenia patients vs. unmedicated healthy humans measured with [11c] gsk215083 pet. *Psychiatry Research: Neuroimaging*, 295:111007, 2020.

[43] Ansel T Hillmer, I Esterlis, Jean-Dominique Gallezot, F Bois, Ming-Qiang Zheng, Nabeel Nabulsi, Shu-Fei Lin,

RL Papke, Yiyun Huang, Osama Sabri, et al. Imaging of cerebral $\alpha4\beta2$* nicotinic acetylcholine receptors with (-)-[18f] flubatine pet: Implementation of bolus plus constant infusion and sensitivity to acetylcholine in human brain. *Neuroimage*, 141:71–80, 2016.

[44] Stephen R Baldassarri, Ansel T Hillmer, Jon Mikael Anderson, Peter Jatlow, Nabeel Nabulsi, David Labaree, Kelly P Cosgrove, Stephanie S O'Malley, Thomas Eissenberg, Suchitra Krishnan-Sarin, et al. Use of electronic cigarettes leads to significant beta2-nicotinic acetylcholine receptor occupancy: evidence from a pet imaging study. *Nicotine and Tobacco Research*, 20(4):425–433, 2018.

[45] Mika Naganawa, Nabeel Nabulsi, Shannan Henry, David Matuskey, Shu-Fei Lin, Lawrence Slieker, Adam J Schwarz, Nancy Kant, Cynthia Jesudason, Kevin Ruley, et al. First-in-human assessment of 11c-lsn3172176, an m1 muscarinic acetylcholine receptor pet radiotracer. *Journal of Nuclear Medicine*, 62(4):553–560, 2021.

[46] M Aghourian, C Legault-Denis, JP Soucy, P Rosa-Neto, S Gauthier, A Kostikov, P Gravel, and MA Bedard. Quantification of brain cholinergic denervation in alzheimer's disease using pet imaging with [18 f]-feobv. *Molecular psychiatry*, 22(11):1531–1538, 2017.

[47] Marc-Andre Bedard, Meghmik Aghourian, Camille Legault-Denis, Ronald B Postuma, Jean-Paul Soucy, Jean-François Gagnon, Amélie Pelletier, and Jacques Montplaisir. Brain cholinergic alterations in idiopathic rem sleep behaviour disorder: a pet imaging study with 18f-feobv. *Sleep medicine*, 58:35–41, 2019.

[48] Marian Galovic, Adam Al-Diwani, Umesh Vivekananda, Francisco Torrealdea, Kjell Erlandsson, Tim D Fryer, Young T Hong, Benjamin A Thomas, Colm J McGinnity, Evan Edmond, et al. In vivo nmda receptor function in people with nmda receptor antibody encephalitis. *medRxiv*, pages 2021–12, 2021.

[49] Marian Galovic, Kjell Erlandsson, Tim D Fryer, Young T Hong, Roido Manavaki, Hasan Sari, Sarah Chetcuti, Benjamin A Thomas, Martin Fisher, Selena Sephton, et al. Validation of a combined image derived input function and venous sampling approach for the quantification of [18f] ge-179 pet binding in the brain. *Neuroimage*, 237:118194, 2021.

[50] Colm J McGinnity, Alexander Hammers, Daniela A Riaño Barros, Sajinder K Luthra, Paul A Jones, William Trigg, Caroline Micallef, Mark R Symms, David J Brooks, Matthias J Koepp, et al. Initial evaluation of 18f-ge-179, a putative pet tracer for activated n-methyl d-aspartate receptors. *Journal of Nuclear Medicine*, 55(3):423–430, 2014.

[51] Kelly Smart, Sylvia ML Cox, Stephanie G Scala, Maria Tippler, Natalia Jaworska, Michel Boivin, Jean R Séguin, Chawki Benkelfat, and Marco Leyton. Sex differences in [11 c] abp688 binding: a positron emission tomography study of mglu5 receptors. *European journal of nuclear medicine and molecular imaging*, 46(5):1179–1183, 2019.

[52] Jonathan M DuBois, Olivier G Rousset, Jared Rowley, Manuel Porras-Betancourt, Andrew J Reader, Aurelie Labbe, Gassan Massarweh, Jean-Paul Soucy, Pedro Rosa-Neto, and Eliane Kobayashi. Characterization of age/sex and the regional distribution of mglur5 availability in the healthy human brain measured by high-resolution [11 c] abp688 pet. *European journal of nuclear medicine and molecular imaging*, 43(1):152–162, 2016.

[53] Martin Nørgaard, Vincent Beliveau, Melanie Ganz, Claus Svarer, Lars H Pinborg, Sune H Keller, Peter S Jensen, Douglas N Greve, and Gitte M Knudsen. A high-resolution in vivo atlas of the human brain's benzodiazepine binding site of gabaa receptors. *NeuroImage*, 232:117878, 2021.

[54] Jean-Dominique Gallezot, Beata Planeta, Nabeel Nabulsi, Donna Palumbo, Xiaoxi Li, Jing Liu, Carolyn Rowinski, Kristin Chidsey, David Labaree, Jim Ropchan, et al. Determination of receptor occupancy in the presence of mass dose:[11c] gsk189254 pet imaging of histamine h3 receptor occupancy by pf-03654746. *Journal of Cerebral Blood Flow & Metabolism*, 37(3):1095–1107, 2017.

[55] Marc D Normandin, Ming-Qiang Zheng, Kuo-Shyan Lin, N Scott Mason, Shu-Fei Lin, Jim Ropchan, David Labaree, Shannan Henry, Wendol A Williams, Richard E Carson, et al. Imaging the cannabinoid cb1 receptor in humans with [11c] omar: assessment of kinetic analysis methods, test–retest reproducibility, and gender differences. *Journal of Cerebral Blood Flow & Metabolism*, 35(8):1313–1322, 2015.

[56] Deepak Cyril D'Souza, Jose A Cortes-Briones, Mohini Ranganathan, Halle Thurnauer, Gina Creatura, Toral Surti, Beata Planeta, Alexander Neumeister, Brian Pittman, Marc D Normandin, et al. Rapid changes in cannabinoid 1 receptor availability in cannabis-dependent male subjects after abstinence from cannabis. *Biological psychiatry: cognitive neuroscience and neuroimaging*, 1(1):60–67, 2016.

[57] Mohini Ranganathan, Jose Cortes-Briones, Rajiv Radhakrishnan, Halle Thurnauer, Beata Planeta, Patrick Skosnik, Hong Gao, David Labaree, Alexander Neumeister, Brian Pittman, et al. Reduced brain cannabinoid receptor availability in schizophrenia. *Biological psychiatry*, 79(12):997–1005, 2016.

[58] Alexander Neumeister, Marc D Normandin, James W Murrough, Shannan Henry, Christopher R Bailey, David A Luckenbaugh, Keri Tuit, Ming-Qiang Zheng, Isaac R Galatzer-Levy, Rajita Sinha, et al. Positron emission tomography shows elevated cannabinoid cb 1 receptor binding in men with alcohol dependence. *Alcoholism: Clinical and Experimental Research*, 36(12):2104–2109, 2012.

[59] Tatu Kantonen, Tomi Karjalainen, Janne Isojärvi, Pirjo Nuutila, Jouni Tuisku, Juha Rinne, Jarmo Hietala, Valtteri Kaasinen, Kari Kalliokoski, Harry Scheinin, et al. Interindividual variability and lateralization of μ-opioid receptors in the human brain. *NeuroImage*, 217:116922, 2020.
