# Supplementary material for: Network spreading and local biological vulnerability in amyotrophic lateral sclerosis
Source: Commun Biol. 2025 Aug 4;8:1153. doi: 10.1038/s42003-025-08561-3 (PMC12322078; doi:10.1038/s42003-025-08561-3)
Supplement: Supplementary file 2 — Description of Additional Supplementary Files [file 42003_2025_8561_MOESM2_ESM.pdf]

Description of Additional Supplementary Files

File name: Supplementary Data 1

Description: Gene-enrichment

File name: Supplementary Data 2

Description: Gene-correlation
