# Supplementary material for: Network spreading and local biological vulnerability in amyotrophic lateral sclerosis
Source: Commun Biol. 2025 Aug 4;8:1153. doi: 10.1038/s42003-025-08561-3 (PMC12322078; doi:10.1038/s42003-025-08561-3)
Supplement: Supplementary file 5 — Reporting summary [file 42003_2025_8561_MOESM5_ESM.pdf]

nature portfolio

Corresponding author(s): Bratislav Misic

Last updated by author(s): 2025/07/13

Reporting Summary

Nature Portfolio wishes to improve the reproducibility of the work that we publish. This form provides structure for consistency and transparency in reporting. For further information on Nature Portfolio policies, see our Editorial Policies and the Editorial Policy Checklist.

Statistics

For all statistical analyses, confirm that the following items are present in the figure legend, table legend, main text, or Methods section.

n/a	Confirmed	
☐	☒	The exact sample size (*n*) for each experimental group/condition, given as a discrete number and unit of measurement
☐	☒	A statement on whether measurements were taken from distinct samples or whether the same sample was measured repeatedly
☐	☒	The statistical test(s) used AND whether they are one- or two-sided<br>*Only common tests should be described solely by name; describe more complex techniques in the Methods section.*
☐	☒	A description of all covariates tested
☐	☒	A description of any assumptions or corrections, such as tests of normality and adjustment for multiple comparisons
☐	☒	A full description of the statistical parameters including central tendency (e.g. means) or other basic estimates (e.g. regression coefficient) AND variation (e.g. standard deviation) or associated estimates of uncertainty (e.g. confidence intervals)
☐	☒	For null hypothesis testing, the test statistic (e.g. *F*, *t*, *r*) with confidence intervals, effect sizes, degrees of freedom and *P* value noted<br>*Give P values as exact values whenever suitable.*
☒	☐	For Bayesian analysis, information on the choice of priors and Markov chain Monte Carlo settings
☒	☐	For hierarchical and complex designs, identification of the appropriate level for tests and full reporting of outcomes
☐	☒	Estimates of effect sizes (e.g. Cohen's *d*, Pearson's *r*), indicating how they were calculated

Our web collection on statistics for biologists contains articles on many of the points above.

Software and code

Policy information about availability of computer code

Data collection	Data was retrieved from the Canadian ALS Neuroimaging Consortium (CALSNIC) dataset (http://calsnic.org). The dataset comprises data from individuals diagnosed with possible, probable, or definite ALS, according to the revised El Escorial Criteria, alongside data from healthy controls. Gene data comes from Allen Brain atlas and is processed using Abagen software.
Data analysis	All codes used to perform the analyses are available on GitHub at https://github.com/netneurolab/Farahani_ALS and on Zenodo at https://zenodo.org/records/15865751 (DOI: 10.5281/zenodo.15865751). Data was analyzed using Python 3.11, MATLAB R2023b, netneurotools v0.2, neuromaps, and ABAnotate software.

For manuscripts utilizing custom algorithms or software that are central to the research but not yet described in published literature, software must be made available to editors and reviewers. We strongly encourage code deposition in a community repository (e.g. GitHub). See the Nature Portfolio guidelines for submitting code & software for further information.

Data

Policy information about availability of data

All manuscripts must include a data availability statement. This statement should provide the following information, where applicable:

- Accession codes, unique identifiers, or web links for publicly available datasets
- A description of any restrictions on data availability
- For clinical datasets or third party data, please ensure that the statement adheres to our policy

All codes used to perform the analyses are available on GitHub at https://github.com/netneurolab/Farahani_ALS and on Zenodo at https://zenodo.org/records/15865751 (DOI: 10.5281/zenodo.15865751). T1w data of ALS patients and healthy control participants come from the CALSNIC dataset (https://calsnic.org/). Brain networks used in the paper can be found at: https://github.com/netneurolab/Farahani_ALS/tree/main/data/Network. Parcellation files (Schaefer and JHU atlases) can be found here: https://github.com/netneurolab/Farahani_ALS/tree/main/data/parcellations.

Research involving human participants, their data, or biological material

Policy information about studies with human participants or human data. See also policy information about sex, gender (identity/presentation), and sexual orientation and race, ethnicity and racism.

Reporting on sex and gender	The CALSNIC dataset comprises both male and female participants, with a relatively balanced representation of sexes (192 individuals with ALS, 70 of whom are female; 175 healthy controls, including 96 females). The main aim of the study was to investigate the overall reliance of ALS atrophy patterns on different brain annotations, independent of sex and age of the affected individuals. To minimize confounding by these variables when generating mean ALS atrophy maps, we regressed out both sex and age effects before analysis.
Reporting on race, ethnicity, or other socially relevant groupings	Participants in the CALSNIC dataset are recruited across North America. ALS patients are included in CALSNIC if they are diagnosed with sporadic or familial ALS, and meet the revised El Escorial research criteria for possible, probable, probable-laboratory supported, or definite ALS.
Population characteristics	We have regressed out age and sex effects when calculating the ALS atrophy maps; as CALSNIC is a multi-site dataset, we also regressed out the effect of imaging center.
Recruitment	Patients are included in CALSNIC if they are diagnosed with sporadic or familial ALS, and meet the revised El Escorial research criteria for possible, probable, probable-laboratory supported, or definite ALS.
Ethics oversight	All participants gave written informed consent, and the CALSNIC data collection was approved by the health research ethics boards at each of the participating sites. All ethical regulations relevant to human research participants were followed.

Note that full information on the approval of the study protocol must also be provided in the manuscript.

Field-specific reporting

Please select the one below that is the best fit for your research. If you are not sure, read the appropriate sections before making your selection.

☒ Life sciences ☐ Behavioural & social sciences ☐ Ecological, evolutionary & environmental sciences

For a reference copy of the document with all sections, see nature.com/documents/nr-reporting-summary-flat.pdf

Life sciences study design

All studies must disclose on these points even when the disclosure is negative.

Sample size	ALS data comes from 192 patients and healthy control data comes from 175 participants.
Data exclusions	Exclusion criteria included the presence of neurological illness other than ALS. Longitudinal data of subjects were not included in this study and we only focused on analyzing the baseline data of participants.
Replication	The findings reported here are inferred using only the CALSNIC dataset.
Randomization	No randomization was done in this study.
Blinding	Blinding is not relevant to this study.

Reporting for specific materials, systems and methods

We require information from authors about some types of materials, experimental systems and methods used in many studies. Here, indicate whether each material, system or method listed is relevant to your study. If you are not sure if a list item applies to your research, read the appropriate section before selecting a response.

Materials & experimental systems

n/a	Involved in the study
☒	☐ Antibodies
☒	☐ Eukaryotic cell lines
☒	☐ Palaeontology and archaeology
☒	☐ Animals and other organisms
☒	☐ Clinical data
☒	☐ Dual use research of concern
☒	☐ Plants

Methods

n/a	Involved in the study
☒	☐ ChIP-seq
☒	☐ Flow cytometry
☐	☒ MRI-based neuroimaging

Plants

Seed stocks	N/A
Novel plant genotypes	N/A
Authentication	N/A

Magnetic resonance imaging

Experimental design

Design type	3D T1w MRI data
Design specifications	Only the baseline images (one T1w data per subject) are used in this study.
Behavioral performance measures	There were no performance measures during the MRI acquisition session. The clinical manifestations of ALS patients were acquired independently.

Acquisition

Imaging type(s)	T1w structural MRI data
Field strength	3.0 T
Sequence & imaging parameters	The CALSNIC T1w protocol was acquired at 1 mm isotropic spatial resolution to provide seamless harmonization and integration across the imaging centers. Each center's T1w imaging sequence parameters can be found here: https://www.medrxiv.org/content/10.1101/2020.07.10.20142679v2.
Area of acquisition	Whole brain scan

Diffusion MRI ☐ Used ☒ Not used

Preprocessing

Preprocessing software	All T1-weighted MRI data were pre-processed using the Medical Imaging Network Common dataform toolkit of the Montreal Neurological Institute, publicly available at https://github.com/BIC-MNI/minc-tools.
Normalization	All T1w images were first linearly and then nonlinearly registered to an average template (MNI152).
Normalization template	Montreal Neurological Institute (MNI152-2009c) standard space
Noise and artifact removal	The following steps have been performed: (i) denoising; (ii) intensity inhomogeneity correction; and (iii) image intensity normalization according to a linear histogram matching algorithm.
Volume censoring	N/A

Statistical modeling & inference

Model type and settings	The contribution of brain connectomes in patterning the ALS atrophy was investigated.
Effect(s) tested	We tested if ALS-related atrophy can be better explained by using the empirical brain networks compared to rewired null networks that randomize network topology, including both degree-preserving and degree and edge length-preserving nulls.

Specify type of analysis: ☐ Whole brain ☐ ROI-based ☒ Both

Anatomical location(s): N/A

Statistic type for inference (See Eklund et al. 2016)	N/A
Correction	False Discovery Rate (FDR), when applicable.

Models & analysis

n/a	Involved in the study
☒	☐ Functional and/or effective connectivity
☐	☒ Graph analysis
☐	☒ Multivariate modeling or predictive analysis

Graph analysis	This study utilized a range of brain networks, including structural connectivity, functional connectivity, gene co-expression similarity, laminar similarity, metabolic similarity and receptor similarity across brain regions. The contributions of each of these networks in the propagation of the disease were investigated. With an atrophy map and an underlying brain connectome, we also identified putative disease epicenters using either epidemiological data-driven methods or computational models.
Multivariate modeling and predictive analysis	A susceptible-infected-removed (SIR) dynamical model was developed to mimic the course of ALS-atrophy spreading while considering the brain structural connectome as the underlying foundation for the spread of pathogenic agents. Partial least squares (PLS) was used to relate clinical manifestations of the disease into the cortical epicenter maps.
